# Repeat ablation strategies in atrial fibrillation patients with durably isolated pulmonary veins: insights from the Netherlands Heart Registration

**DOI:** 10.1007/s12471-026-02021-1

**Published:** 2026-02-11

**Authors:** Federico Tancredi Magni, Michelle Samuel, Bart A. Mulder, Michelle van der Stoel, Rutger J. Hassink, Serge A. Trines, Michiel J. B. Kemme, Jippe C. Balt, Pepijn H. van der Voort, Justin G. L. M. Luermans, Jonas S. S. G. de Jong, Yuri Blaauw

**Affiliations:** 1 Department of Cardiology, University Medical Center Groningen, University of Groningen, Groningen, The Netherlands; 2 Department of Epidemiology, University Medical Center Groningen, University of Groningen, Groningen, The Netherlands; 3 Department of Medicine, Dalhousie University, Halifax, Canada; 4 Netherlands Heart Registration, Utrecht, The Netherlands; 5 Department of Cardiology, University Medical Center Utrecht, Utrecht, The Netherlands; 6 Department of Cardiology, Heart Lung Center, Leiden University Medical Center, Leiden, The Netherlands; 7 Department of Cardiology, Amsterdam University Medical Center, Amsterdam, The Netherlands; 8 Department of Cardiology, St. Antonius Hospital, Utrecht, The Netherlands; 9 Department of Cardiology, Catharina Hospital, Eindhoven, The Netherlands; 10 Department of Cardiology, Cardiovascular Research Institute Maastricht (CARIM), Maastricht University Medical Center (MUMC+), Maastricht, The Netherlands; 11 Department of Cardiology, Hospital Onze Lieve Vrouwe Gasthuis, Amsterdam, The Netherlands

**Keywords:** Atrial fibrillation, Catheter ablation, Repeat ablation, Durably isolated pulmonary veins, Registry

## Abstract

**Background and aims:**

In 15–40% of patients undergoing repeat ablation for AF recurrence, all pulmonary veins (PVs) are durably isolated. Currently, there is limited evidence on the appropriate treatment strategy for these patients. We aimed to characterize and compare the effectiveness of different re-ablation strategies.

**Methods:**

All patients referred for repeat AF ablation with all PVs durably isolated at 8 hospitals in the Netherlands were included [Netherlands-Heart-Registration (NHR); 2016–2019]. NHR data were were used to determine the presence of PV-reconnection, the ablation strategy used, and the outcome of ablation (atrial arrhythmia recurrence > 30 sec.). The effectiveness of ablation strategies was assessed with multivariable Cox models.

**Results:**

Of 2311 repeat AF ablations performed, 274 (11.9%) patients had all PVs durably isolated. Median age was 66 (IQR:58–70) years, 44.2% women, 45.6% had persistent/long-standing-persistent AF. In 33 (12.0%) patients, no ablation was performed. A single ablation strategy was performed most often (41.2%). Posterior wall ablation (58.4%) was performed most often, followed by PV-antralization (26.3%). Over 2.0 (1.0–3.3) years, 147 (59.8%) patients had an atrial arrhythmia recurrence, and 30 (12.7%) patients had another repeat AF ablation within 1 year. After multivariable adjustment, no difference in atrial-arrhythmia recurrences was detected between individual ablation strategies, number of strategies performed, and type of atrial-arrhythmia (*p* > 0.05 for all). Left-atrial-volume-index was associated with a higher recurrence-risk [aHR 1.03(95%CI 1.01–1.05)].

**Conclusion:**

In patients with durably isolated PVs, a high proportion experienced recurrence of atrial arrhythmias, with no difference in recurrence rates between different re-ablation strategies.

**Supplementary Information:**

The online version of this article (10.1007/s12471-026-02021-1) contains supplementary material, which is available to authorized users.

## What’s new?

- The present analysis of the Netherlands Heart Registration shows that durably isolated pulmonary veins are found in 11.9% of patients undergoing a repeat procedure for atrial fibrillation following previous PVI-only procedures.
- In a large group of patients with durably isolated pulmonary veins, a wide variety of ablation strategies was employed, ranging from no ablation to various combinations.
- In patients with durably isolated pulmonary veins, no significant difference in effectiveness was observed between these strategies after a median of 2 years of follow-up.

## Introduction

Despite advances in ablation strategies and technologies, atrial fibrillation (AF) recurs in 25–50% of patients after pulmonary vein isolation (PVI) [1–6]. Pulmonary vein (PV) reconnection is commonly found during repeat ablation, where re-isolation remains the primary goal [1, 7–9]. However, 15–40% of patients undergoing repeat ablation show all PVs to be durably isolated on electrophysiological mapping [1, 10–13]. Despite the high incidence, limited evidence is available, and no expert consensus exists on the optimal ablation strategy in patients with durably isolated PVs. This has led to a wide array of operator-dependent approaches such as linear ablation lesions, low-voltage area ablation, and trigger ablation [10, 14]. Therefore, we conducted a nationwide comparison of the effectiveness and safety of various real-world ablation strategies used during repeat AF ablation in patients with durably isolated PVs in the Netherlands, utilizing data from the Netherlands Heart Registration.

## Methods

### Study design and patient population

This retrospective, multicenter cohort study was conducted at 8 Dutch centers using data from the Netherlands Heart Registration (NHR), a nationwide quality-monitoring registry that includes all AF ablations since 2013 [15]. We identified all repeat ablations performed between 2016–2019. The registry provides detailed demographic, medical history, and procedural data [16]. Institutional review board approval was obtained (MEC‑U W19.270), and informed consent was waived.

Inclusion criteria were: (1) paroxysmal or persistent AF treated with PVI-only ablation; (2) documented AF recurrence (ECG or Holter) after the first procedure; and (3) first repeat ablation between 2016–2019 with confirmed durable PV isolation. Of note, only one patient had longstanding persistent AF at baseline; due to the insufficient sample size for separate analysis, this patient was grouped with those classified as having persistent AF. Exclusion criteria included prior surgical ablation, additional ablation lesions (except CTI), or repeat procedures for atrial tachycardia/flutter.

### Data collection

The NHR database provided comprehensive data on all ablation procedures, including demographics, medical history, and prior ablations. In 2022, the registry was expanded to include additional variables such as hypertension, CAD, CVA/TIA, diabetes, PV reconnection status, repeat ablation strategy, follow-up dates, antiarrhythmic drug use, arrhythmia recurrence details, and arrhythmia type. This extended dataset was collected by eight centers.

Follow-up began at the first repeat ablation and was conducted per local protocols, typically involving visits at 3, 6, and 12 months, then annually. During visits, symptoms, adverse events, and arrhythmia recurrence assessed via ECG or Holter monitoring were registered. Additional visits occurred as needed for symptomatic recurrence. The last recorded follow-up, recurrence, or further ablation marked the endpoint for survival analysis.

### Repeat AF ablations

All centers assessed PVI durability during repeat procedures using a multipolar mapping catheter and 3D electro-anatomical mapping. Once PV isolation was confirmed, the electrophysiologist determined the ablation strategy, as documented in the procedure report.

**Fig. 1:**
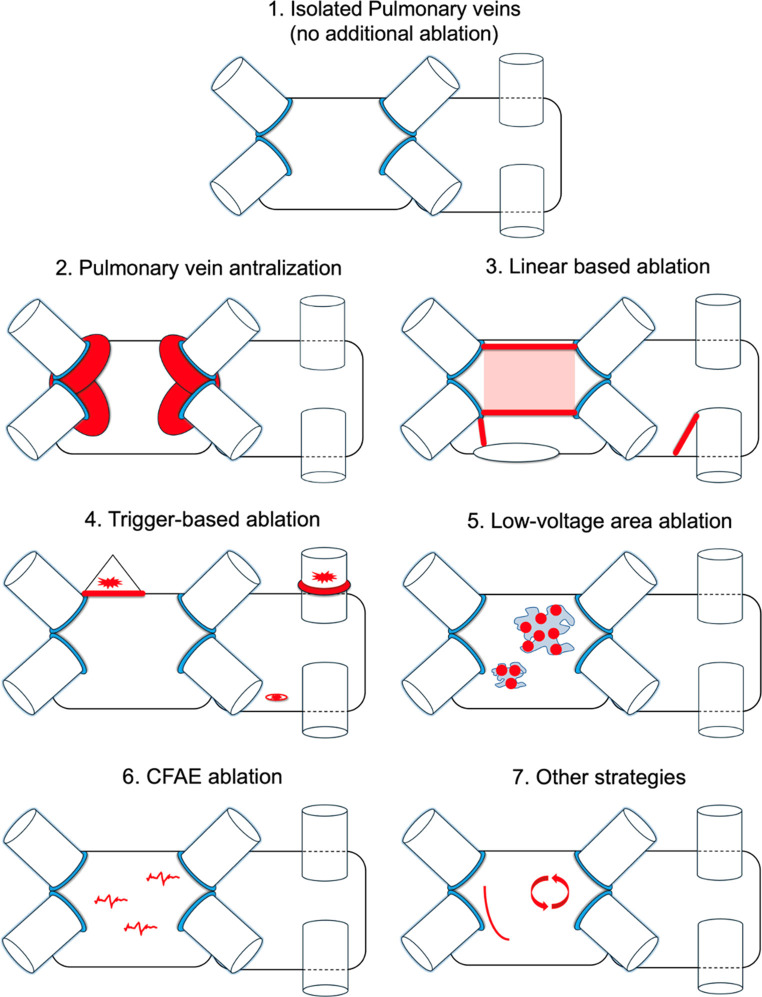
Overview of investigated ablation strategies. Visual representation of the individual ablation strategies investigated in the study: 1) Durably isolated Pulmonary Veins (no additional ablation); 2) Pulmonary Vein Antralization; 3) Linear-Based Ablation: roof line, inferior line, posterior wall [both as box made using roof and inferior lines, as well as whole posterior wall (shaded red area)], cavo-tricuspid isthmus line, mitral isthmus line; 4) Trigger-Based Ablation: superior vena cava, coronary sinus, left atrial appendage, other triggers; 5) Low-Voltage Area Ablation; 6) CFAE (Complex Fractionated Atrial Electrograms) Ablation; 7) Other Strategies: vein of Marshall ablation, rotor ablation

Strategies were categorized as follows (Fig. 1): (1) No additional ablation; (2) PV antralization (more antral PVI lesions); (3) linear ablation (roof line, inferior line, posterior box, mitral isthmus, CTI line); (4) trigger ablation (e.g., SVC, LAA, coronary sinus); (5) low-voltage area ablation; (6) complex fractionated atrial electrogram ablation; (7) other strategies (e.g., vein of Marshall or rotor ablation).

### Study endpoints

The main objective of the study was to assess atrial arrhythmia-free survival following a repeat ablation procedure, with an arrhythmia recurrence defined as any symptomatic or asymptomatic episode of AF, atrial tachycardia, or atrial flutter lasting at least 30 s—as confirmed by a 12-lead ECG or a 24-hour Holter monitor—and occurred after a 90-day blanking period following the initial repeat ablation.

### Statistical analysis

Continuous variables were reported as medians with interquartile ranges and compared using the Mann-Whitney U test. Categorical variables were presented as counts and percentages, compared using Chi-square or Fisher’s exact test, as appropriate. Patients were grouped by recurrence status and AF type (paroxysmal vs. persistent).

Ablation strategy effectiveness was assessed using multivariable Cox proportional hazards models, adjusted for age, sex, BMI, AF type, diabetes, CAD, and hypertension. Cox models compared individual strategies, the number of strategies used, and the presence of persistent AF. Time-to-recurrence was analyzed using Kaplan-Meier curves and log-rank tests. All strategies were evaluated independently and in combination, with interaction analyses performed to assess their combined effects. Results were reported as hazard ratios (HR) with 95% confidence intervals (CI); significance was set at *p* < 0.05. Analyses were conducted in STATA v18 (StataCorp, TX, USA).

## Results

### Patient population and prior ablation procedures

Between January 2016 and December 2019, 2,311 repeat ablations were performed (100% radiofrequency) across eight centers in the Netherlands. Of these, 274 procedures (12%) met the inclusion criteria of AF recurrence with durably isolated PVs (ESM Figure S1A).

Baseline characteristics are detailed in Tab. 1. The median age was 66 years (IQR 58–70), 44.2% were women, and the median CHA_2_DS_2_-VASc score was 2.0 (IQR 1.0–3.0). Paroxysmal AF was present in 54.4% and persistent AF in 45.6%. Patient characteristics were similar between AF types, except for higher rates of coronary artery disease (11.5% vs. 4.1%), hypertension (62.1% vs. 45.5%), and greater weight [89 kg (80–98) vs. 81 kg (72–96)] in persistent AF (all *p* < 0.05; see ESM Tab S1A).

**Table 1:** Baseline characteristics

Characteristic	*n* (%)/median (IQR)
Age, years	66 (58–70)
Women	121 (44.2%)
BMI (kg/m^2^)	27.17 (24.41–29.77)
*Type of AF*	
Paroxysmal	124 (54.4%)
Persistent	104 (45.6%)
Previous CVA/TIA	23 (8.4%)
Diabetes	16 (5.8%)
CAD (medication, PCI, or CABG)	19 (7.0%)
Hypertension	141 (51.8%)
Serum creatinine	82 (71–95)
LVEF (%)	55 (55–55)
LA size (ml/m2)	39 (31–46)
*CHA*_*2*_*DS*_*2*_*-VAS*_*C*_* score*	2 (1–3)
0	34 (15.0%)
1	44 (19.3%)
2	61 (26.9%)
3	54 (23.8%)
≥ 4	34 (15.0%)
*Preoperative mitral valve insufficiency*	
None/Mild	181 (89.6%)
Moderate	21 (10.4%)
Severe	0 (0.0%)

*BMI* body mass index, *AF* atrial fibrillation, *CVA* cerebrovascular accident, *TIA* transient ischemic accident, *CAD* coronary artery disease, *PCI* percutaneous coronary intervention, *CABG* coronary artery bypass graft, *LVEF* left ventricular ejection fraction, *LA* left atrium

### Ablation strategies for repeat ablation in patients with durably isolated PVs

**Fig. 2:**
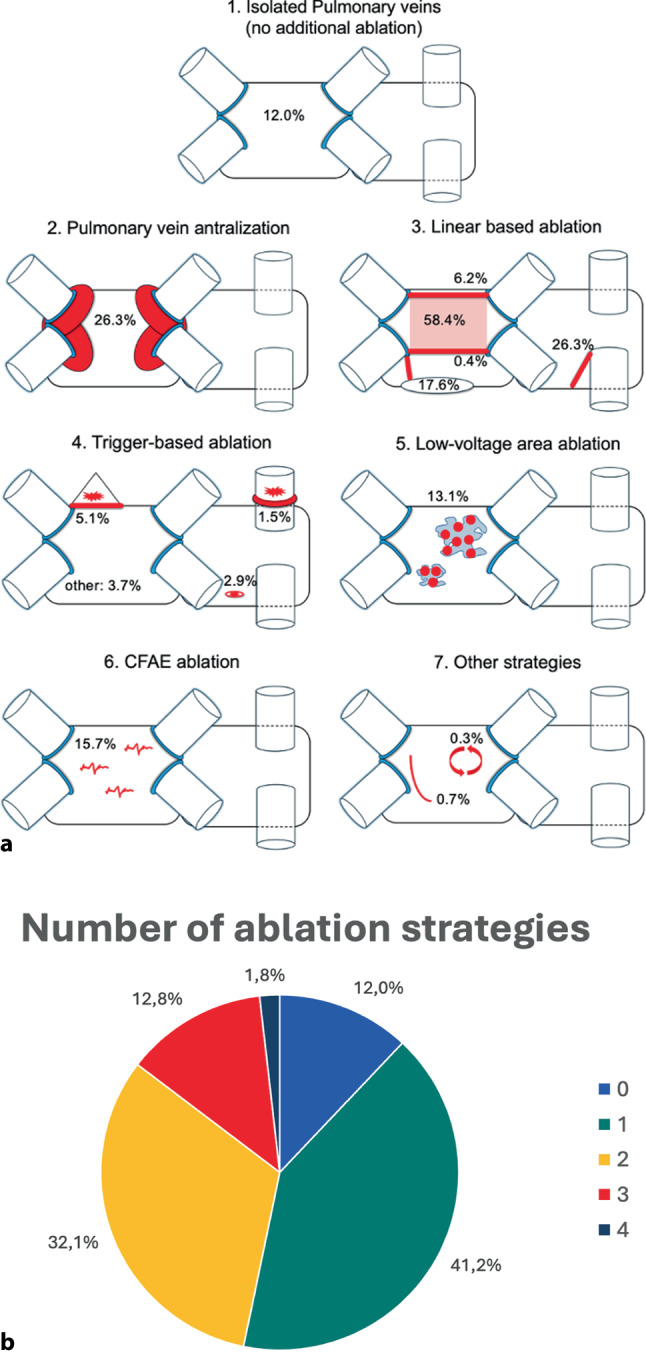
Distribution of individual ablation strategies and number of ablation strategies performed. **a** Visual representation of the percentage of patients who received each specific ablation strategy; **b** Pie chart showing the percentage distribution of the number of ablation strategies/combinations performed among all included patients

Figure 2 shows the distribution of ablation strategies among the 274 patients with durably isolated PVs. No additional ablation was performed in 12.0% of cases. A single ablation strategy was performed most often (41.2%), followed by two (32.1%), three (12.8%), and four (1.8%) strategies.

Posterior box ablation was the most common approach (58.4%), followed by antralization of the PVs (26.3%). Roof line ablation alone was performed in 17 patients (6.2%), and an inferior line alone in 1 patient (0.4%). Posterior box creation via combined roof and inferior lines was used in 34.3% of patients, accounting for 58.8% of all posterior wall ablation cases. A mitral isthmus line was performed in 48 patients (17.6%), and CTI ablation in 72 (26.3%). Trigger ablation was performed in 33 patients (12.0%), targeting the following sites: superior vena cava in 14 (5.1%), left atrial appendage in 4 (1.5%), coronary sinus in 8 (2.9%), and other sites in 10 (3.7%). Low-voltage area ablation was done in 36 patients (13.1%) and CFAE ablation in 43 (15.7%).

Comparision based on type of AF showed posterior box ablation was significantly more common in patients with persistent AF (*p* < 0.05; ESM Table S1A), who also more frequently underwent a higher number of ablation strategies compared to paroxysmal AF (*p* < 0.05).

### Follow-up and effectiveness

Patients were monitored for a median of 2.0 years (IQR 1.0–3.3). During follow-up, 142 patients (52.8%) experienced atrial arrhythmia recurrence > 90 days post-procedure, and 30 patients (12.7%) underwent another ablation within 12 months (Tab. 2). At the last follow-up, 141 patients (57.3%) were still using anti-arrhythmic drugs.

**Table 2:** Repeat ablation outcomes

Outcome	Total (*n* = 274)
AAD use at last known follow-up	141 (57.32%)
Atrial arrhythmia recurrence (> 3 months)	142 (52.79%)
AFL/AT	46 (17.10%)
Paroxysmal AF	47 (17.47%)
Persistent AF	49 (18.22%)
Median time to AF recurrence	222 (125–449) days
Repeat ablation within < 1 year	30 (12.71%)

*AAD* anti-arrhythmic drug, *AF* atrial fibrillation, *AFL/AT* atrial flutter or atrial tachycardia

**Fig. 3:**
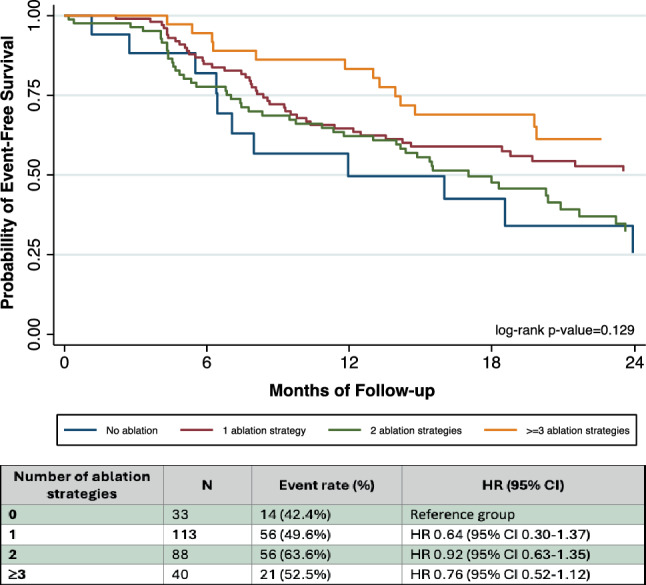
Kaplan-Meier curve for number of strategies performed. Kaplan-Meier survival curve illustrating the probability of atrial-arrhythmia-free survival over a 24-month follow-up period for patients with durably isolated pulmonary veins undergoing different numbers of ablation strategies during repeat ablation. ^a^Adjusted for age, sex, BMI, paroxysmal AF, diabetes, CAD, hypertension, year of repeat ablation, and number of ablation strategies performed; ^b^Outcome: AF recurrence

Multivariable Cox regression adjusted for age, sex, BMI, AF type, diabetes, CAD, and hypertension found no individual ablation strategy independently associated with arrhythmia recurrence (*p* > 0.05 for all; ESM Figure S2A). Subgroup analysis by AF type also showed no difference in recurrence rates [HR 1.001, 95% CI 0.67–1.51]. Similarly, the number of strategies used did not affect recurrence when compared to no ablation (*p* > 0.05; Fig. 3). Sensitivity analyses confirmed the robustness of these results.

Left atrial volume index was the only independent predictor of arrhythmia recurrence in multivariable analysis [adjusted HR 1.03, 95% CI 1.01–1.05; ESM Figure S3A].

### Complications

A total of 8 patients (3.5%) experienced complications post-ablation (Tab. 3). Two (0.9%) had cardiac tamponade. One patient had a major vascular complication, and five (2.2%) experienced minor vascular issues, primarily venous bleeding at the femoral puncture site.

**Table 3:** Complications of repeat ablation procedures

Complication	*N *(%)
Cardiac tamponade	2 (0.87)
Major vascular complications	1 (0.44)
Minor vascular complications	5 (2.18)

## Discussion

This multicenter NHR study examined ablation strategies in patients with durably isolated PVs undergoing repeat AF ablation. A wide range of strategies is being used, with posterior wall ablation being the most common. However, no significant differences in arrhythmia recurrence were observed between strategies, including no additional ablation, regardless of AF type (paroxysmal or persistent).

### Incidence of durably isolated PVs during repeat AF ablation

Previous studies have shown a variable incidence of durably isolated PVs in patients with AF recurrence presenting for repeat AF ablation, with reported rates ranging from 15–40%, depending on patient population, operator expertise, and ablation technique [1, 10–12]. The percentage of patients with isolated pulmonary veins is slightly lower than previously reported in the literature. Our data is based on a real-world registry in which ablation was performed using various techniques by a broad range of operators with differing levels of experience. This may explain the discrepancy compared to other studies, which were often conducted in expert centers, either within or outside the framework of standardized ablation protocols—for example, as part of a multicenter randomized controlled trial.

The incidence of durable PVI increases with the number of previous PVI procedures [17, 18]. A sub-analysis of the FIRE AND ICE trial examining findings at repeat ablation procedures indicated a higher incidence of durably isolated PVs following cryoballoon compared to radiofrequency ablation (21.9% vs. 17.3%) [19]. However, a recent study by De Potter et al. reported a much higher incidence of 62% after index ablation using CLOSE-guided ablation, which involves precise delivery of contact-force guided point-by-point radiofrequency ablation [11]. Emerging technologies, such as pulsed field ablation, may further improve lesion durability [20]. Data from two recently published large multicenter registries, the EU-PORIA registry and the MANIFEST registry, observed that 38% and 45.5% of patients, respectively, had durably isolated PVs at repeat ablation after initial PVI with pulsed field ablation [21, 22]. Considering this is a first-generation technology, with the improvement and development of novel ablation technologies and strategies, the number of patients with durably isolated PVs during repeat procedures is likely to increase.

### Current ablation practices in patients with durably isolated PVs

The presence of durably isolated PVs in many patients with recurrent AF suggests that arrhythmogenic sources lie outside the PVs. While extra-PV ablation strategies have shown benefits in select subgroups, no single approach has proven superior to PVI. This uncertainty explains the lack of consensus on the best ablation strategy for treating these patients, suggesting that an individualized approach may be necessary.

In the recent retrospective PARTY-PVI study, Benali et al. compared various ablation strategies during repeat ablation for AF in 367 patients with durably isolated PVs from 39 centers [10]. Most patients were males (67%) with persistent AF recurrence (56.4%). Only one ablation strategy (54.5%) was commonly used, followed by two or three (37.1% and 6.5%). Similar to our findings, they observed no significant difference in AF-free survival across strategies, with LA size being the only independent predictor of recurrence. Notably, Benali et al. grouped similar strategies to boost statistical power, but did not assess the effects of individual strategies, report the overall incidence of durably isolated PVs, or specify how many patients received no additional ablation. Ackmann et al. reported a similar analysis using a contemporary ablation cohort. Durable PVI was observed during remapping in only 20.1% [13]. A range of ablation strategies was employed in patients who presented with isolated pulmonary veins. They also demonstrate that no specific ablation approach yields superior outcomes over another.

Recently, preliminary findings from the ASTRO-AF study were presented [14]. This multicenter, prospective, randomized study compared substrate modification and left atrial appendage isolation in 161 patients with durably isolated PVs [14]. They found no statistically significant difference in AF/AT recurrence at one year between the two ablation strategies. Of note, more than half of the patients had undergone more than one prior ablation procedure. Due to futility, the study was prematurely terminated after randomizing 63% of the planned patient population.

In our study, posterior wall isolation was the predominant strategy (58.4%) employed in patients with durably isolated PVs. The posterior wall is widely accepted as a major extra-PV harbor for AF triggers and drivers, partly attributed to the shared embryological development with the PVs [23, 24]. However, conclusive evidence regarding its efficacy beyond PVI alone remains inconsistent and inconclusive, with currently available data showing contrasting results [25, 26].

Few studies have assessed outcomes of repeat ablation in patients with durably isolated PVs. In our cohort, atrial arrhythmia recurred in 52.8% of patients. The PARTY-PVI study by Benali et al. reported a 43.3% recurrence rate at two years, with no significant differences between various ablation strategies or their combinations [10]. Similarly, the ASTRO-AF study found one-year recurrence rates of 48.3% for low-voltage area ablation and 44.5% for empirical left atrial appendage isolation, again with no significant difference [14]. De Pooter et al. observed a 39% recurrence rate at one year using empirical trigger ablation (e.g., superior vena cava isolation or PV antralization) and substrate ablation (linear lesions at the roof, mitral isthmus, or anterior wall) [11]. A small single-center series reported comparable outcomes using diverse approaches such as extra-pulmonary trigger ablation, CFAE ablation, and linear lesions [27, 28]. Unlike our study, none of these investigations included a control group of patients who underwent no additional re-ablation.

Our study, like others, shows that outcomes of AF ablation in the setting of isolated pulmonary veins are suboptimal, and no conclusions can be drawn regarding a preferred ablation strategy. Future studies are needed to provide more clarity, starting with the use of pulsed-field ablation (PFA). Evidence for the optimal strategy should come from a well-powered multicenter randomized controlled trial. Potential approaches may include ablation guided by high-density (functional) substrate mapping [29, 30]. Promising new techniques, such as spatiotemporal dispersion ablation [31], also warrant further investigation.

### Limitations

The main limitation of the current study is that it is an observational study. There was no control group, and the study was not randomized. Also, our study is subject to selection bias, as the strategy and choice to perform additional ablation were determined by the operator at the time of the procedure In addition, detailed procedural data, such as ablation settings and exact ablation locations, were unavailable, and the execution of ablation strategies is operator dependent. This limits the ability to assess strategy-specific effects.

AF recurrence detection was not standardized, possibly underestimating recurrence; nevertheless, recurrence rates were high, suggesting that AF recurrence is very common in this subset of patients.

Pulsed field ablation was not yet utilized in the current analysis. It is possible that if PFA had been used as the ablation technique instead of radiofrequency, the outcome of the analysis might have been different. It should be emphazised that identification of these patients is only possible during repeat procedures, making large-scale inclusion difficult. Nonetheless, through the NHR, we identified one of the largest cohorts of patients with durably isolated PVs.

## Conclusion

In this large cohort of patients with AF recurrence despite durably isolated PVs, we examined current repeat ablation practices across the Netherlands. We observed a wide variety of strategies, from no ablation to multiple combinations, with no significant differences in effectiveness, regardless of AF type (paroxysmal or persistent). Prospective, randomized studies are needed to determine whether, and which, additional ablation strategies beyond PVI offer benefit in this patient population and to assess the value of personalized treatment approaches.

## Supplementary Information

ESM1: Supplementary material 1
