# Supplementary material for: Repeat ablation strategies in atrial fibrillation patients with durably isolated pulmonary veins: insights from the Netherlands Heart Registration
Source: Neth Heart J. 2026 Feb 11;34(3):107–16. doi: 10.1007/s12471-026-02021-1 (PMC12920987; doi:10.1007/s12471-026-02021-1)
Supplement: Supplementary file 1 — ESM1: Supplementary material 1 [file 12471_2026_2021_MOESM1_ESM.docx]

**Appendix**

**Table S1A. Comparison of Paroxysmal vs Persistent AF patients**

| Variable | Paroxysmal AF  (n=124) | Persistent AF  (n=104) | p-value |
| --- | --- | --- | --- |
| Age, years | 65 (58-70) | 66 (59.5-71) | 0.193 |
| Women | 63 (50.81%) | 40 (38.46%) | 0.062 |
| Height, cm | 178 (168-185) | 177 (170-184) | 0.697 |
| Weight, kg | 81 (72-96) | 89 (80-98) | 0.0081 |
| Serum creatinine | 90 (69-94) | 84.5 (74-97) | 0.089 |
| LVEF (%) | 55 (55-55) | 55 (55-55) | 0.053 |
| LA size | 39 (31-46) | 38.5 (32-45) | 0.588 |
| CHA2DS2-VASC score  0  1  2  3  ≥4 | 2 (1-3)  21 (17.01%)  23 (18.70%)  35 (28.46%)  28 (22.76%)  16 (13.01%) | 2 (1-3)  13 (12.75%)  21 (20.59%)  26 (25.49%)  24 (23.53%)  18 (17.65%) | 0.30  0.1691 |
| Preoperative mitral valve insufficiency  None/Mild  Moderate | 99 (92.52%)  8 (7.48%) | 80 (86.02%)  13 (13.98%) | 0.135 |
| Previous CVA/TIA | 10 (8.06%) | 8 (7.69%) | 0.917 |
| Diabetes | 5 (4.03%) | 11 (10.58%) | 0.054 |
| CAD (medication, PCI, or CABG) | 5 (4.07%) | 12 (11.54%) | 0.033 |
| Hypertension | 56 (45.53%) | 64 (62.14%) | 0.013 |
| Ablations |  |  |  |
| Antral ablation  Left PVs  Right PVs | 27 (24.77%)  17 (13.71%)  21 (17.07%) | 32 (32.00%)  20 (19.23%)  24 (23.08%) | 0.246  0.26  0.258 |
| Antral ablation of left PV & CTI | 6 (4.84%) | 4 (3.85%) | 0.715 |
| Posterior box ablation | 68 (48.23%) | 73 (51.77%) | 0.017 |
| Roof line ablation only  (between RSPV & LSPV) | 13 (10.48%) | 6 (5.77%) | 0.200 |
| Inferior line ablation only (between RIPV & LIPV) | 1 (0.81%) | 3 (2.88%) | 0.234 |
| Mitral isthmus line ablation | 18 (14.52%) | 25 (24.27%) | 0.062 |
| CTI ablation | 32 (25.81%) | 29 (27.88%) | 0.724 |
| Low voltage area ablation | 14 (11.29%) | 16 (15.38%) | 0.362 |
| CFAE | 17 (13.71%) | 13 (12.50%) | 0.788 |
| Other ablation strategies  Rotor ablation  Vein of Marshall | 1 (0.81%)  1 (0.81%) | 0 (0%)  0 (0%) | 0.429 |
| Number of ablation methods  0  1  2  3  4 | 1 (1-2)  18 (14.52%)  55 (44.35%)  39 (31.45%)  11 (8.87%)  1 (0.81%) | 2 (1-2)  6 (5.77%)  43 (41.35%)  35 (33.65%)  16 (15.38%)  4 (3.85%) | 0.0118  0.068 |

*Values are displayed as either median and inter-quartile range or frequency and percentage of total.*


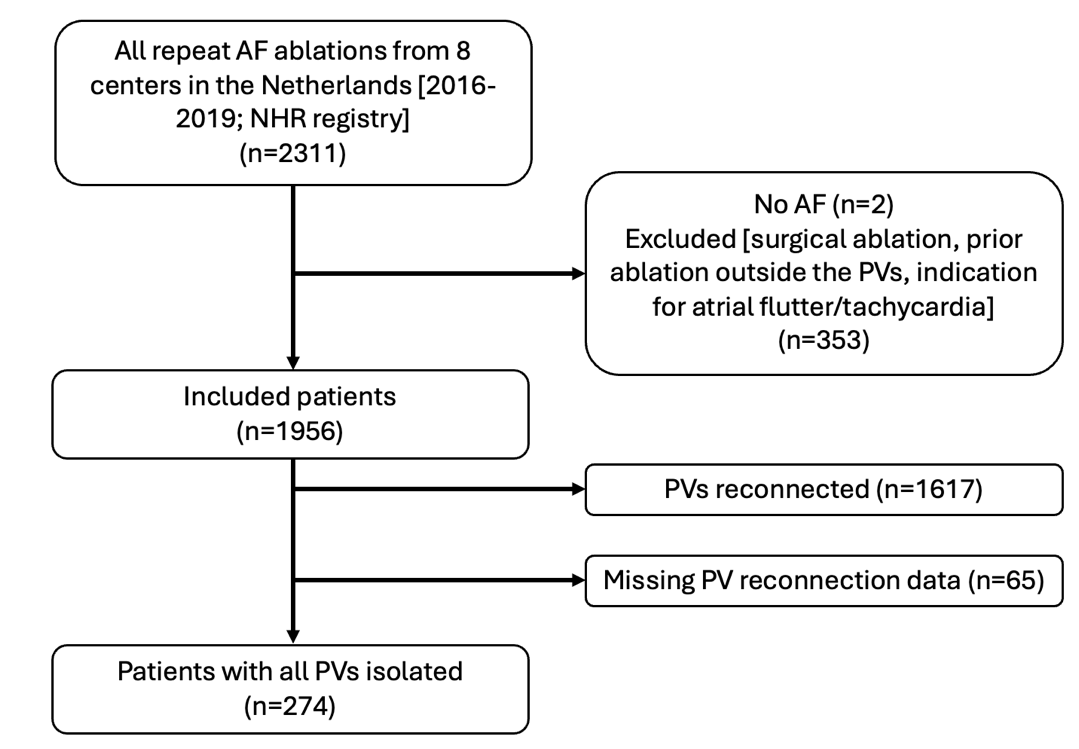


**Figure S1A. Flowchart study population.**

Overview of the included and excluded patients. This figure shows the selection process of patients undergoing repeat AF ablations from eight centers in the Netherlands (2016-2019; NHR). Out of 2311 patients, 1956 eligible patient were identified after excluding those with no AF (n=2) and those with surgical ablation, prior ablation outside the PVs, or an indication for atrial flutter/tachycardia (n=353). Among eligible patients, 1617 had reconnected pulmonary veins (PVs), 65 had missing PV reconnection data, and a total of 274 patients had all PVs isolated, which were included in the final analyses.

^a Adjusted for age, sex, BMI, paroxysmal AF, diabetes, CAD, hypertension, year repeat ablation was performed^

^b Outcome: AF recurrence.^

**Figure S2A. Forest plot for effectiveness of ablation methods.**

Forest plot displaying the outcomes of a multivariate Cox regression analysis, comparing the hazard ratios (HR) on rates of atrial arrhythmia recurrence among different ablation strategies relative to performing no re-ablation at all. The cox regression analysis was conducted following multivariable adjustment for age, sex, body-mass index, paroxysmal AF, diabetes, CAD, and hypertension

**Figure S3A. Forest plot for predictors of AF recurrence**

Forest plot displaying the outcomes of a multivariate Cox regression analysis, comparing the hazard ratios (HR) for recurrence of atrial fibrillation between different factors, adjusted for ablation strategy.
